# Docteur! regardez ma langue!

**DOI:** 10.11604/pamj.2017.27.75.11632

**Published:** 2017-06-01

**Authors:** Amina Kissou, Badr Eddine Hassam

**Affiliations:** 1 Department of Dermatology Ibn Sina Hospital Centre, Rabat, Morocco

**Keywords:** Langue, noire, villeuse, Tongue, black, villous

## Image en médecine

La langue noire villeuse est une condition bénigne. C'est une modification de la couleur de l'aspect de la langue qui devient noire, sale, avec des papilles filiformes allongées. Plusieurs facteurs sont impliqués: tabagisme, consommation excessive de café / thé, mauvaise hygiène bucco-dentaire, névralgie du trijumeau et certains médicaments. Nous rapportons un nouveau cas.

**Figure 1:**
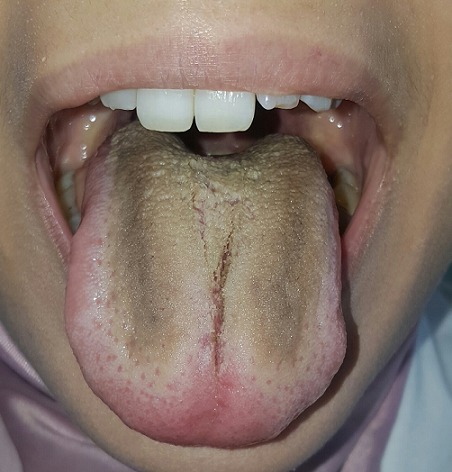
Langue noire villeuse

